# Brief Report: Nasal colonization with Staphylococcus aureus and methicillin resistant Staphylococcus aureus among community-dwelling older adults with comorbidities seeking follow-up medical care in Central Sri Lanka

**DOI:** 10.1099/acmi.0.000724.v3

**Published:** 2024-05-21

**Authors:** Ifaaz Iqbal, Zeenath Sabri, Adithya Illangasinghe, Ashini Isurindi, Rashmi Jayakodi, Wasana Jayasekara, Kaveesha Jayarathna, Nonduni Jayasinghe, Mekhala Ishani, Ishan Jayasekara, Nelum Handapangoda, Dilrukshi Menike, Rasadanie Dissanayake, Asela Ekanayake, Veranja Liyanapathirana

**Affiliations:** 1 Faculty of Medicine, University of Peradeniya, Peradeniya, Sri Lanka; 2 Department of Microbiology, Faculty of Medicine, University of Peradeniya, Peradeniya, Sri Lanka

**Keywords:** colonization, elderly, MRSA, older adults, *Staphylococcus aureus*

## Abstract

Older adults are more severely affected by infections caused by drug-resistant bacteria including Methicillin-Resistant *Staphylococcus aureus* (MRSA). We aimed to identify the MRSA colonization rates and associated factors among older adults aged more than 65-years-old. Among the 309 recruited, 152 (49.2 %) were males. Self-collected nasal swabs were used to isolate *Staphylococcus aureus* and MRSA with routine microbiological methods. *Staphylococcus aureus* was isolated from 36 (11.7 %) participants while 11 (3.6 %) were colonized with MRSA. We identified a significant association between the male sex and MRSA colonization (*P*=0.028, Chi-square test). However, this needs careful interpretation given the smaller number of outcome events. Other factors studied had no statistically significant association with MRSA colonization.

## Data Summary

The datasets generated and analysed during the current study is avaliable in supplementary material with the online version of this article.

## Introduction

The presence of comorbidities, their treatment and immunosenescence make older adults more susceptible to infections. Frailty, accidents, and falls also increase the risk of infections. Further, an increased level of antibiotic resistance in isolates causing infections among this demography is also noted [1].

Methicillin-Resistant *Staphylococcus aureus* (MRSA) is a major contributor to antimicrobial resistance (AMR)-associated deaths [2]. Therefore, we selected the colonization rate with MRSA as a surrogate for the colonization burden of AMR among older adults. Further, colonization with MRSA in itself is linked to a higher mortality rate among middle-aged and older adults [3].

This study was conducted with the aim of determining the colonization rate with *Staphylococcus aureus* and MRSA among older adults, and factors associated with such colonization.

## Methods

This was a cross-sectional study conducted among adults >65-years-old attending the Teaching Hospital Peradeniya, Sri Lanka for clinic visits between 21 November 2022 to 28 November 2022. Consecutive clinic attendees were recruited after obtaining informed written consent. Those with respiratory symptoms, bleeding disorders, nasal bleeding, nasal polyps, or nasal surgery, and those receiving intra-nasal treatment were excluded. An interviewer-administered data collection sheet was used to gather demographic details and associated factors for colonization with *Staphylococcus aureus* or MRSA as listed in Table 1.

**Table 1.** Description of the study population and factors associated with colonization with *Staphylococcus aureus* and MRSA

Variables		Overall	No. of total participants		Significance	No. of total participants		Significance
			Participants not colonized with *S. aureus*	Participants colonized with *S. aureus*		Participants not colonized with MRSA	Participants colonized with MRSA	
Age	<75	216 (69.9 %)	190 (88.0 %)	26 (12.0 %)	0.747	209 (96.8 %)	7 (3.2 %)	0.739
	=>75	93 (30.1 %)	83 (89.2 %)	10 (10.8 %)		89 (95.7 %)	4 (4.3 %)	
Sex	Male	152 (49.2 %)	133 (87.5 %)	19 (12.5 %)	0.647	143 (94.1 %)	9 (5.9 %)	**0.028**
	Female	157 (50.8 %)	140 (89.2 %)	17 (10.8 %)		155 (98.7 %)	2 (1.3 %)	
Ethnicity	Sinhalese	267 (86.4 %)	237 (88.8 %)	30 (11.2 %)	0.084	257 (96.3 %)	10 (3.7 %)	0.329
	Sri Lankan Moor	29 (9.4 %)	27 (93.1 %)	2 (6.9 %)		29 (100 %)	0 (0)	
	Tamil	11 (3.6 %)	8 (72.7 %)	3 (27.3 %)		10 (90.0 %)	1 (9.1 %)	
	Burger	2 (0.6 %)	1 (50.0 %)	1 (50.0 %)		2 (100 %)	0 (0)	
Previous hospital admission within a year	Yes	105 (34.0 %)	95 (90.5 %)	10 (9.5 %)	0.403	102 (97.1 %)	3 (2.9 %)	0.755
	No	204 (66.0 %)	178 (87.3 %)	26 (12.7 %)		196 (96.1 %)	8 (3.9 %)	
Surgical interventions within a year	Yes	21 (6.8 %)	20 (95.2 %)	1 (4.8 %)	0.487	21 (100 %)	0 (0)	1.000
	No	288 (93.2 %)	253 (87.8 %)	35 (12.2 %)		277 (96.2 %)	11 (3.8 %)	
Antibiotics within the preceding 3 months	Yes	29 (9.4 %)	27 (93.1 %)	2 (6.9 %)	0.749	29 (100 %)	0 (0)	0.470
	No	213 (68.9 %)	186 (87.3 %)	27 (12.7 %)		203 (95.3 %)	10 (4.7 %)	
	Don’t know	67 (21.7 %)	60 (89.6 %)	7 (10.4 %)		66 (98.5 %)	1 (1.5 %)	
Number of children in the household	=<3	90 (29.1 %)	79 (87.8 %)	11 (12.2 %)	1.000	88 (97.8 %)	2 (2.2 %)	0.611
	>3	6 (1.9 %)	6 (100 %)	0 (0)		6 (100.0 %)	0 (0)	
	0	213 (68.9 %)	188 (88.3 %)	25 (11.7 %)		204 (95.8 %)	9 (4.2 %)	
Number of occupants in the household	=<5	254 (82.2 %)	222 (87.4 %)	32 (12.6 %)	0.264	245 (96.5 %)	9 (3.5 %)	1.000
	>5	55 (17.8 %)	51 (92.7 %)	4 (7.3 %)		53 (96.4 %)	2 (3.6 %)	
Sleep in separate room/ not	No	25 (8.1 %)	22 (88.0 %)	3 (12.0 %)	1.000	24 (96.0 %)	1 (4.0 %)	1.000
	Separate room	284 (91.9 %)	251 (88.4 %)	33 (11.6 %)		274 (96.5 %)	10 (3.5 %)	
Pets in the house	Yes	128 (41.4 %)	116 (90.6 %)	12 (9.4 %)	0.294	124 (96.9 %)	4 (3.1 %)	1.000
	No	181 (58.6 %)	157 (86.7 %)	24 (13.3 %)		174 (96.1 %)	7 (3.9 %)	
Association with farm animals	Yes	13 (4.2 %)	12 (92.3 %)	1 (7.7 %)	1.000	13 (100 %)	0 (0)	1.000
	No	296 (95.8 %)	261 (88.2 %)	35 (11.8 %)		285 (96.3 %)	11 (3.7 %)	
More than one co-morbidity present	Yes	231 (74.8 %)	204 (88.3 %)	27 (11.7 %)	0.972	224 (97.0 %)	7 (3.0 %)	0.478
	No	78 (25.2 %)	69 (88.5 %)	9 (11.5 %)		74 (94.9 %)	4 (5.1 %)	
Vegan	Yes	28 (9.1 %)	25 (89.3 %)	3 (10.7 %)	1.000	25 (89.3 %)	3 (10.7 %)	0.067
	No	281 (90.9 %)	248 (88.3 %)	33 (11.7 %)		273 (97.2 %)	8 (2.8 %)	
Current alcohol consumption	Yes	33 (10.7 %)	27 (81.8 %)	6 (18.2 %)	0.247	30 (90.9 %)	3 (9.1 %)	0.101
	No	276 (89.3 %)	246 (89.1 %)	30 (10.9 %)		268 (97.1 %)	8 (2.9 %)	
Current smoking	Yes	19 (6.1 %)	17 (89.5 %)	2 (10.5 %)	1.000	18 (94.7 %)	1 (5.3 %)	0.508
	No	290 (93.9 %)	256 (88.3 %)	34 (11.7 %)		280 (96.6 %)	10 (3.4 %)	
Immunomodulators/Chemotherapeutic agent Used	Yes	27 (8.7 %)	24 (88.9 %)	3 (11.1 %)	0.304	27 (100 %)	0 (0)	0.635
	No	280 (90.6 %)	248 (88.6 %)	32 (11.4 %)		269 (96.1 %)	11 (3.9 %)	
	Don’t know	2 (0.6 %)	1 (50.0 %)	1 (50.0 %)		2 (100 %)	0 (0)	

Participants were instructed to insert the swab into the nasal canal and rotate the swab around the nasal wall for 10 s and repeat the procedure in the other nostril. The swab was transported to the Department of Microbiology, Faculty of Medicine, University of Peradeniya, Sri Lanka within 2 h of collection. Swabs were processed with routine microbiological methods. Briefly, the swabs were incubated overnight in 6.5 % sodium chloride in nutrient broth and 10 µl of the broth was plated on Manitol Salt Agar and Blood agar plates. Potential colonies based on appearance were identified with Gram-stain, catalase test, coagulase test (slide and tube) and DNAse test. *Staphyococcus aureus* isolates were Gram-positive cocci which were catalase-, coagulase- and DNAse-positive. MRSA isolates were identified with cefoxitin (30 µg) sensitivity testing using the disc diffusion method [4]. Antibiotic susceptibility testing was also done for erythromycin (15 µg), clindamycin (2 µg), ciprofloxacin (5 µg), gentamycin (10 µg), and inducible clindamycin resistance was also tested [4].

Ethics approval was obtained from Ethics Review Committee of Faculty of Medicine, University of Peradeniya (2022/EC/SP/12).

Percentages were calculated to identify colonization rates. Descriptive statistics were used to describe the demographic details. Age, sex, ethnicity, hospital admission within a year, surgical interventions within a year, antibiotic use within the preceding 3 months, number of children in the household, number of occupants in the household, sleeping arrangement (in separate room/ not), pets in the house, association with farm animals, presence of more than one comorbidity, veganism, current alcohol consumption, current smoking, smoking and being on immunomodulators/chemotherapy drugs were analysed to determine the possible association with *Staphylococcus aureus* and MRSA colonization with Chi-square test and Fisher’s Exact test. *P* value<0.05 was taken to be statistically significant.

## Results and discussion

Of the 309 participants 36 (11.7 %) were colonized with *Staphylococcus aureus* and 11 (3.6 %) were colonized with MRSA. A UK study with 342 participants aged above 65 years revealed that 120 (35.08 %) were colonized with *Staphylococcus aureus* with 54 (15.78 %) being positive for MRSA [5]. A Brazilian study with 120 healthy adults above 60 years found 21 (17.8 %) to be colonized with *Staphylococcus aureus* and four (3.39 %) with MRSA [6]. A Sri Lankan study with 502 participants from all age groups revealed that 101 (20.1 %) were colonized with *Staphylococcus aureus* and 31 (6.2 %) were colonized with MRSA. This study was done among patients who were being hospitalized for various ailments [7].

It’s clear that our percentage was comparatively lower than other population studies done locally and globally. Our population of participants was coming from the community. Even though all of them had comorbidities, the number who were hospitalized within the previous 3 months and underwent surgical procedures was relatively lower. We assessed the nasal colonization only. However, of the individual sites that are available, nose is taken as the better single site and many studies have used this site alone [8,9]. Many of the studies done abroad have considered older adults who are dwelling in care facilities. Participants of the current study were community dwelling, where some of the risk factors for colonization with antibiotic-resistant bacteria are minimal. Most of our study participants lived in households with fewer than five members and had their own rooms to sleep, these could explain the lower colonization rates as overcrowding is one of the main factors leading to MRSA colonization.

Ten of the 11 MRSA isolates were resistant to erythromycin, two to clindamycin and three each to ciprofloxacin and gentamicin. Of the 25 methicillin-sensitive *Staphylococcus aureus* (MSSA) isolates ten were resistant to erythromycin, two to clindamycin, ten to ciprofloxacin and one to gentamycin. Considering betalactams, macrolides, quinolones and aminoglycosides, four MRSA isolates were resistant to three or more classes of antibiotics while none of the MSSA were resistant to three or more classes of antibiotics. This indicates that despite the presence of comorbidities, the presence of multi-drug resistance is lower among the study group.

Nineteen (12.5 %) out of 152 males and 17 (10.8 %) out of 157 females were colonized with *Staphylococcus aureus*. None of the factors studied were associated significantly with colonization with *Staphylococcus aureus*. Nine (5.9 %) out of 152 males and two (1.3 %) out of 157 females were colonized with MRSA (*P*=0.028, Chi-square test). None of the other factors studied were associated with colonization with MRSA (Table 1).

No association was seen between sex and *Staphylococcus aureus* colonization. Even though previous studies have shown an association with sex, results of Grundmann *et al.* were similar to ours [5]. But even though not significant, our percentages supported previous studies like Andersen *et al.* with males having higher colonization than females [10]. We were able to find a significant association between sex and MRSA colonization with males showing a higher percentage than females (9 [5.9 %] vs 2 [1.3 %], *P*=0.028). This builds on the existing evidence of many researchers including Hasanpour *et al*. [11]. Anatomical and physiological (body hair, and distribution of body hair), and behavioural traits may be the reason for the gender difference in nasal colonization with MRSA.

## Limitations

No significant association was identified between any of the other factors studied and colonization with *Staphylococcus aureus* or MRSA. This could be due to the lesser number of colonization events or due to socio-cultural differences. There may have been a recall bias as data were taken from patients. We noted no ethnic differences in the colonization rates, contrary to some of the previous studies. However, the number of participants coming from ethnic minorities was relatively fewer. While our data may be considered negative data, we believe it is important to present this to highlight the deviations from the expected results.

We only used nasal swabs for sampling and the sample was self-collected. However, self-collected nasal swabs have been recommended as a cost-effective method for surveillance studies [9,12].

## supplementary material

10.1099/acmi.0.000724.v3 Uncited Supplementary Material 1.

## Abbreviations

AMR: antimicrobial resistance
MRSA: methicillin resistant *Staphylococcus aureus*
MSSA: methicillin sensitive *Staphylococcus aureus*
